# Associations of Perilipin 3 with Insulin Resistance in Arab Adults with Type 2 Diabetes

**DOI:** 10.1155/2021/4791915

**Published:** 2021-11-02

**Authors:** Amani Alghamdi, Dalal Z. Alhotti, Shaun Sabico, Omar S. Al-Attas, Nasser M. Al-Daghri

**Affiliations:** ^1^Biochemistry Department, College of Science, King Saud University, Riyadh, Saudi Arabia; ^2^Biomarkers of Chronic Diseases, Biochemistry Department, College of Science, King Saud University, Riyadh, Saudi Arabia

## Abstract

**Objective:**

The role of lipid metabolism disorders in the pathogenesis of T2DM has been recognized. Lipid droplets (LDs) are dynamic organelles that store lipids. Perilipin 3 (PLIN3) is one of the five LD coat proteins that is relatively understudied as compared to other LDs. This study is aimed at determining levels of PLIN3 among adults with varying levels of obesity and insulin resistance to determine metabolic associations of PLIN3. *Methodology*. A total of 280 Saudi adults (*n* = 127 males; *n* = 153 females) were randomly recruited and divided into three groups according to their body mass index (BMI) and fasting glucose levels: healthy and lean (HL), obese and T2DM (OD), or obese and nondiabetic (OND). Lipid profiles, fasting glucose levels, insulin, and perilipin 3 levels were measured.

**Results:**

Circulating PLIN3 was significantly lower in the OD group [8.3 ng/mL (1.2–22.5; *p* < 0.001)] than the HL group [23.1 ng/mL (6.2–39.1; *p* < 0.001)]. Triglycerides, total cholesterol, glucose, and insulin levels were inversely correlated with PLIN3 in all subjects. Lastly, glucose, insulin, and total cholesterol cumulatively predict circulating levels of PLIN3 by as much as 11% of the variances perceived (*p* < 0.001).

**Conclusion:**

Circulating PLIN3 is significantly associated with insulin resistance markers and maybe a promising candidate as a protective biomarker for T2DM.

## 1. Introduction

Diabetes mellitus (DM) is a group of metabolic disorders characterized and identified by the presence of hyperglycemia. The heterogeneous etiopathology includes defects in insulin secretion, insulin action, or both and disturbances of carbohydrate, fat, and protein metabolism [1]. According to the eighth edition of the International Diabetes Federation (IDF)'s Diabetes Atlas, one type 2 DM (T2DM) patient dies worldwide every 8 seconds [2]. Roughly 425 million adults live with T2DM today, and by 2045, 693 million people are expected to have become affected [3]. T2DM is an adult onset diabetes that is the type afflicting around 90–95% of DM patients [4, 5]. The kingdom of Saudi Arabia is not immune to this disease and has one of the highest T2DM populations in the world, with 7 million Saudis living with DM another 3 million having prediabetes [6].

In T2DM patients, roughly 8 out of 10 are either overweight or obese [7]. Obesity disrupts the functions of adipose tissue. Normally, adipose tissue secretes retinol binding protein 4 (RBP4), adiponectin, resistin, pigment epithelium-derived factor (PEDF), and hundreds of adipokines linked to metabolism and insulin sensitivity [8]. The lipid droplets (LDs) are surrounded by phospholipid monolayer to shield the natural lipids in the cellular hydrophobic environment. The phospholipid monolayer of LDs enclose toxic lipids and protect the cell from lipotoxicity and oxidative stress. The first identified LD proteins were perilipins (PLINs) and include 5 members in mammals. Generally, PLINs are like gatekeepers for the droplet core and prevent lipolysis through two main mechanisms. In the hyperphosphorylated (stimulated) state, the PLINs recruit the hormone sensitive lipase (HSL) to the surface of LDs, stimulating lipolysis. While in the hypophosphorylated (basal) state, PLINs keep the natural lipids in LD core [9]. PLIN3 has an established role in LD biogenesis [10]. Lipolytic stimulation in primary human myotubes has shown to increase PLIN3 expression in lean individuals than those with T2DM. Other than the LDs, PLIN3 is distributed in tissues of the heart, liver, muscles, intestines, mammary glands, and white adipose [11, 12]. Other known functions of PLIN3 involve mannose 6-phosphate receptor (M6PR) recycling, vision, viral infection, thermogenesis, and immune system [13, 14]. In this study, we aimed to compare for the first time PLIN3 levels among subjects with varying degrees of obesity and insulin resistance and to determine associations of PLIN3 with cardiometabolic indices.

## 2. Methods

### 2.1. Subjects

A total of 280 adult Saudi participants (*n* = 127 males; *n* = 153 females), 25–60 years old, were randomly selected from the master databases of CBCD [15–17]. In brief, the master databases contain clinical information and biological samples of more 10,000 Saudis ages 1-70 recruited from the different primary healthcare centers (PHCs) and schools in Riyadh, Saudi Arabia, last 2010 and 2014. These databases which served as basis for several epidemiologic studies in Riyadh were done in collaboration with the Ministries of Health and Education. Clinical information included medical history based on previous records. T2DM cases were diagnosed based on the World Health Organization (WHO) criteria [4]. Subjects who were known T2DM cases, under anti-T2DM medications, and/or with a fasting blood glucose (FBG) > 7.0 mmol/L, were categorized as T2DM, while those with FBG < 5.6 mmol/L were considered non-T2DM. For the purpose of this study, participants were divided into three groups according to their BMI and T2DM status: a healthy lean (HL) group (*n* = 85), an obese and T2DM (OD) group (*n* = 95), and an obese but nondiabetic (OND) group (*n* = 100). The demographic information, general health status, and medical history of the participants were recorded. The study was approved by the Ethics Committee of the College of Medicine, King Fahad Medical City, Riyadh, Saudi Arabia (IRB 13-094). All subjects with thyroid, cardiac, kidney, liver disease, psychiatric conditions, and/or use of medications were excluded. Furthermore, those who have prediabetes (FBG 5.61-6.99 mmol/l) and underweight (BMI < 18.5 kg/m^2^) were excluded. Anthropometric and clinical measurements included weight (kg), height (cm), waist (cm), hip circumference (cm), and waist-to-hip ratio (WHR). Mean systolic and diastolic blood pressure (mm of Hg) were also recorded. Body mass index (BMI) was calculated as weight (kg) divided by height in squared meters (m^2^) to diagnose obesity (≥30 kg/m^2^) [16].

### 2.2. Blood Sample Collection and Separation

Blood samples were collected from all participants after 8 hours or more of fasting. Vacutainer tubes (Becton Dickinson, Franklin Lakes, NJ, USA) were used to separate serum by slowly inverting the blood samples several times and then leaving them upright at room temperature (22°C) for 30 min to allow clotting. Then, the samples were centrifuged at 3000 rpm for 15 min at 4°C, and the supernatant was carefully separated into aliquots, flash-frozen, and stored at −20°C until analyzed.

### 2.3. Fasting Glucose, Lipids, and Insulin Level Determination

Triglycerides, total cholesterol including HDL and LDL cholesterol levels, fasting glucose levels, and insulin level determinations were carried out routinely [15, 17, 18].

### 2.4. Perilipin 3 Level Determination

A human PLIN3 CLIA kit (Catalogue No. abx494487, Abbexa Ltd., Cambridge, UK) was used to determine PLIN3 in serum through an enzyme-linked immunosorbent assay (ELISA). An antibody specific to PLIN3 was precoated into a 96-well plate, and the standards and samples (with a dilution factor of 5) were added to the wells and incubated. Biotin-conjugated anti-PLIN3 antibody was used for detection. Next, avidin conjugated to horseradish peroxidase (HRP) was added to each microplate well and incubated. After incubation, a 3,3′,5,5′-tetramethylbenzidine (TMB) substrate solution was added. Only in wells that contained PLIN3, biotin-conjugated antibodies, and enzyme-conjugated avidin produced a blue-colored product that changed into yellow after adding an acidic stop solution. The intensity of the yellow color was proportional to the level of PLIN3 bound on the plate. The OD absorbance was measured spectrophotometrically at 450 nm in a microplate reader to calculate the PLIN3 concentration.

### 2.5. Statistical Analysis

Data were analyzed using SPSS software (version 22; IBM Corp., Armonk, NY, USA). Continuous data were presented as mean ± standard deviation (SD) for normal variables, and non-Gaussian variables were presented as median (1st and 3rd) quartiles. Categorical data were presented as frequencies and percentages (%). All continuous variables were checked for normality using the Kolmogorov–Smirnov test. Non-Gaussian variables were log-transformed prior to parametric analysis. One-way ANOVA and the Kruskal–Wallis *H* test were performed to compare mean and median differences in Gaussian and non-Gaussian variables in the groups, and a posthoc Bonferroni analysis performed on the group samples was significantly different. Correlations between variables were calculated using Pearson's and Spearman's correlation analysis. Stepwise linear multiple regression was carried out using log PLIN3 as dependent, and sex, BMI, systolic and diastolic blood pressure, log glucose, total cholesterol, log triglycerides, and log insulin were entered as independent variables to determine significant predictors of PLIN3. Glucose, total cholesterol, and insulin were identified as independent predictors of PLIN3. A *p* value <0.05 was considered statistically significant.

Posthoc power analysis demonstrates that this study had over 90% power with the effect size of 0.24 to detect the difference in the PLIN3 concentration between lean, obese diabetic, and obese nondiabetic subjects at 95% confidence interval.

## 3. Results

### 3.1. Clinical Characteristics of the Participants

A total of 280 subjects (*n* = 127 males; *n* = 153 females) participated in the study. The differences between the HL, OD, and OND groups with regard to the studied characteristics are shown in Table 1. BMI and WHR values in the HL group were significantly lower than the OD group at *p* < 0.001. Similar trends were observed in cases of systolic and diastolic BP, glucose levels, total cholesterol, HDL cholesterol, triglycerides, insulin, and the homeostasis model assessment of insulin resistance (HOMA-IR), where these values in the HL group were significantly lower than in the OD group, at *p* < 0.001. A highly significant difference was found in the level of PLIN3 between the HL group and the OD group at *p* < 0.001. In the case of the OND group, the values for weight, BMI, waist, and hips were significantly higher when compared with the HL group. However, total cholesterol and triglycerides for the OND group were significantly lower than in the OD group. Fasting glucose levels of in the OND group were slightly higher than the HL group but significantly lower than the OND group. Insulin levels and HOMA-IR were significantly lower than in the OD group. PLIN3 concentrations in the OND group were significantly lower than in the HL group but significantly higher than in the OND.

**Figure 1:**
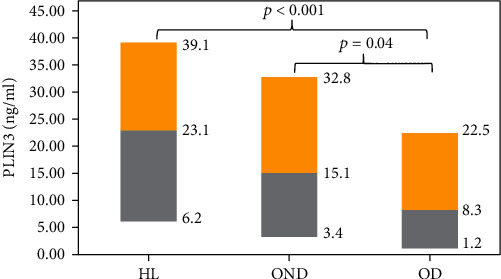
PLIN3 levels in the three groups expressed in terms of median, 1^st^ quartile (gray), and 3^rd^ quartile (yellow).

PLIN3 concentrations in the HL, OD, and OND groups are shown in Figure 1 as median, with the upper yellow parts indicating the 75th percentile and the lower grey parts indicating the 25th percentile. PLIN3 levels in the OD group were significantly lower than in the HL group, while PLIN3 values in the OND group were significantly lower than in the HL group and significantly higher than in the OD group.

### 3.2. Correlations of PLIN3 with Other Parameters

**Figure 2:**
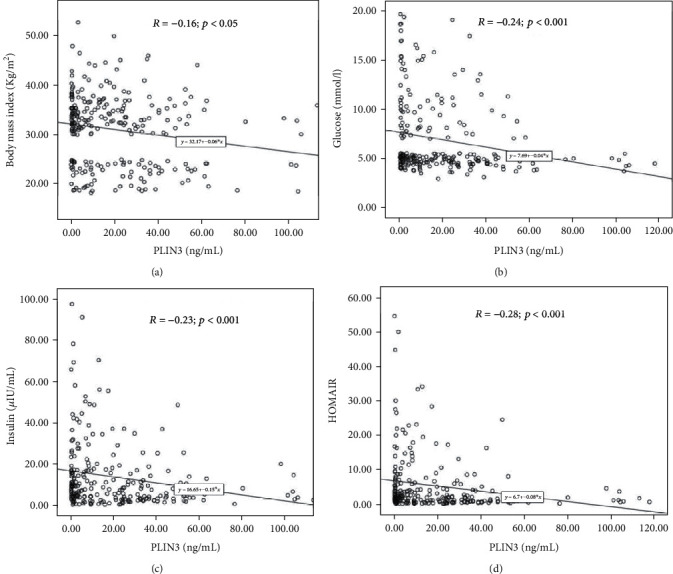
Significant associations of PLIN3 with (a) BMI, (b) glucose, (c) insulin, and (d) HOMA-IR.

The correlations of PLIN3 with the other studied clinical parameters were also determined, and the results are shown in Table 2. Figures 2(a)–2(d) show correlation coefficients for PLIN3 and multiple parameters in the form of scatter plots. The correlation coefficient (*R* = −0.16, *p* < 0.05) showed a significant inverse correlation between PLIN3 and BMI. Similarly, the scatter plot for PLIN3 and glucose showed an inverse correlation (*R* = −0.24, *p* < 0.001) (Figure 2(b)). Insulin and HOMA-IR were also inversely correlated to PLIN3 (*R* = −0.23, *p* < 0.001; *R* = −0.28, *p* < 0.001, respectively), as shown in Figure 2(c). Stepwise linear regression revealed that glucose, insulin, and total cholesterol cumulatively predict circulating levels of PLIN3 by as much as 11% of the variances perceived (*p* < 0.001) (Table 3).

### 3.3. Gender-Specific Variation in Clinical Characteristics in the Studied Groups

Table 4 shows the comparisons of the clinical characteristics of the groups stratified according to sex in this study. They were similar in age and BMI, but the WHR of males was higher than females in all groups. Systolic and diastolic blood pressure was also higher in males compared to females. Fasting glucose levels were similar in males and females, except in the females in the OD group, where slightly higher levels (11.8 ± 3.8 mmol/L) were noted in comparison to males (10.9 ± 3.2 mmol/L). Triglycerides were higher in males than females in all groups, at *p* < 0.001. Insulin and insulin resistance in males were also higher than in females (*p* < 0.001). PLIN3 levels were significantly higher in females than males (*p* < 0.001). Table 3 shows the reverse correlation between BMI and PLIN3 in both genders, but it was significant in males. Insulin and HOMA-IR were significantly reverse correlated with PLIN3 in both males and females.

## 4. Discussion

This study observed that PLIN3 levels in the serum of Saudi subjects who were divided into the three groups i.e. HL, OD, OND. The very limited or contradictory literature on the roles of PLIN3 motivated us to design this study to reveal a link between PLIN3 concentrations in the serum of the three groups with T2DM. Our results showed significant reverse correlations between PLIN3 and insulin resistance indices (glucose, insulin, and HOMA-IR) in all groups. But in each group, the reverse correlation between PLIN3 and insulin resistance indices was not significant, which may be due to the difference in the numbers of participants. BMI and WHR were inversely correlated with PLIN3 levels, as were the total cholesterol, triglycerides, and insulin resistance. The lowest concentration of PLIN3 was observed in the OD group and the highest in the HL group. This observation may be the outcome of a shift of PLIN3 under fatty acid stimulating conditions from cytosol to the surface of LDs, resulting in low levels of PLIN3 in blood of the OD group, while in the HL group, higher amounts of cytosolic PLIN3 in the blood were evident. This seems more logical when we see higher level of glucose, total cholesterol, and insulin in the OD group. These observations agree with the reported literature indicating that PLIN3 is part of LD mitochondria contact sites, promoting efficient transfer of FA from LD to mitochondria for oxidation [19]. The significant and inverse correlations of PLIN3 with BMI, glucose, insulin, and HOMA-IR further support our results. However, the roles of other PLINs as reported by Ali et al. are in contrast with our findings who reported a positive correlation between PLIN1 and insulin sensitivity. They suggested that the high levels of PLIN1 protected LDs from lipolysis and caused cellular starvation, thus activating fasting hormones to elevate glucose levels in the plasma [20]. The inverse correlation between PLIN3 and insulin sensitivity indicates that the PLIN3 level is not directly correlated with lipolysis as perilipin 1 has been; instead, there may be other factors that contribute to lipolysis. One of them may be the presence of perilipin 1 in adipose tissue while PLIN3 in skeletal muscles [21, 22].

Total cholesterol and triglycerides were inversely correlated with PLIN3, which also conflicts with Ali et al. Low levels of total cholesterol and triglycerides with high PLIN3 concentrations may reflect the role of PLIN3 in lipogenesis or due to PLIN3's function as protection for LDs against lipolysis. PLIN3 levels in muscle biopsies from healthy patients have been reported previously to be positively correlated with whole-body oxidative capacity, while PLIN3 knockdown resulted in decreased FA oxidation [23], which can partially explain our findings.

Interestingly, significantly higher PLIN3 levels in females, approximately double that in males, could be due to the presence of PLIN3 in mammary glands and in muscles.

The authors acknowledge some limitations. The cross-sectional design limits our findings to at best, suggestive. Furthermore, medications taken by participants were not included in the analysis, and this covariate can greatly alter results. Dietary intake was also not taken into consideration, and these factors are known to acutely affect lipids and subsequent T2DM risk [24, 25]. Despite these limitations, the study's findings have scientific merit as it sheds new light in the role of PLIN3 in the realm of human metabolism, insulin resistance in particular.

Overall, this study reports a significant inverse association between serum levels of PLIN3 and markers of insulin resistance among Saudi adults with T2DM. These findings will be a valuable addition in the available limited literature on the role of PLIN3 with insulin resistance and diabetes. To the best of our knowledge, this is the 1^st^ such report. However, we recommend future studies with larger number of participants to get more significant data. Furthermore, focus should also be on studying the association of these patterns with lipolysis multiple tissues because of this PLIN function in tissue-specific context.

## 5. Conclusion

Circulating PLIN3 levels are influenced by obesity and T2DM status in Saudi adults, with the lowest levels observed in subjects having both conditions independent of sex. Hence, PLIN3 may be a promising marker for insulin resistance. Mechanistic studies are needed to further explain the physiology behind these associations.

## Figures and Tables

**Table 1:** Clinical characteristics of subjects according to groups.

Parameters	HL	OND	OD	*p* value
N (M/F) (127/153)	85 (41/44)	100 (46/54)	95 (40/55)	
Age (years)	38.8 ± 13.8	39.6 ± 10.5	49.6 ± 10.5^AB^	<0.001
BMI (kg/m^2^)	22.1 ± 2.2	35.9 ± 4.5^A^	34.6 ± 3.6^AB^	<0.001
Waist (cm)	81.5 ± 9.7	103.7 ± 14.1^A^	103.5 ± 14.6^A^	<0.001
Hips (cm)	92.3 ± 8.7	115.5 ± 12.5^A^	109.8 ± 13.4^AB^	<0.001
WHR	0.88 ± 0.12	0.88 ± 0.10	0.93 ± 0.10^AB^	0.005
Systolic BP (mmHg)	113.7 ± 12.9	121.3 ± 11.2^A^	129.1 ± 11.7^AB^	<0.001
Diastolic BP (mmHg)	74.1 ± 6.6	78.3 ± 6.1^A^	81.8 ± 6.8^AB^	<0.001
Glucose (nmol/L)	4.7 ± 0.6	4.9 ± 0.5	11.5 ± 3.6^AB^	<0.001
Total cholesterol (nmol/L)	4.7 ± 0.9	5.0 ± 1.1	5.5 ± 1.5^AB^	<0.001
HDL cholesterol (nmol/L)	1.11 ± 0.3	1.01 ± 0.3^A^	0.94 ± 0.3^A^	<0.001
Triglycerides# (nmol/L)	1.2 (0.8-1.7)	1.5 (1.0-2.4)^A^	2.2 (1.7-3.5)^AB^	<0.001
Insulin# (*μ*IU/mL)	3.8 (1.8-7.4)	9.1 (4.1-14.3)	14.1 (6.1-34)^AB^	<0.001
HOMA-IR#	0.78 (0.3-1.6)	1.9 (0.9-3.3)^A^	7.0 (2.8-16.3)^AB^	<0.001
PLIN3# (ng/mL)	23.1 (6.2-39.1)	15.1 (3.4-32.8)	8.3 (1.2-22.5)^AB^	<0.001

Note: data presented as mean ± SD for normal and median (interquartile range for nonnormal variables). Superscript “A” indicates significant difference from HL, and superscript “B” indicates significance compared to OND. Significant at *p* < 0.05. BMI: body mass index; BP: blood pressure; HDL: high-density lipoprotein; HOMA-IR: homeostasis insulin resistance; PLIN3: perilipin 3; WHR: waist-hip ratio.

**Table 2:** Associations of PLIN3 with studied parameters according to groups.

Parameters	All	HL	OND	OD
*N* (M/F)	280	85	100	95
Age (years)	-0.10	-0.12	-0.01	0.11
BMI (kg/m^2^)	-0.16^∗^	-0.12	0.04	-0.10
Waist (cm)	-0.17^∗∗^	-0.17	-0.05	-0.10
Hips (cm)	-0.10	-0.27^∗^	0.16	0.01
WHR	0.10	0.13	0.10	0.10
Systolic BP (mmHg)	-0.16^∗^	-0.12	-0.18	0.10
Diastolic BP (mmHg)	-0.20^∗∗^	-0.17	-0.24^∗^	0.04
Glucose (mmol/L)	-0.24^∗∗^	-0.18	0.05	-0.20
Total cholesterol (mmol/L)	-0.20^∗∗^	-0.13	-0.15	-0.16
HDL cholesterol (mmol/L)	0.10	0.10	0.11	-0.05
Triglycerides# (mmol/L)	-0.22^∗∗^	-0.11	-0.31^∗∗^	0.01
Insulin# (*μ*IU/mL)	-0.23^∗∗^	-0.13	-0.14	-0.16
HOMA-IR	-0.28^∗∗^	-0.16	-0.16	-0.21

Note: Data presented as coefficient (*R*); ^∗^ indicates significance at 0.05 level; ^∗∗^ indicates significance at 0.01 level. BMI: body mass index; BP: blood pressure; HDL: high-density lipoprotein; HOMA-IR: homeostasis insulin resistance; WHR: waist-hip ratio.

**Table 3:** Independent predictors of PLIN3.

Parameters	B ± SE	*p* values
Log glucose (mmol/l)	−0.46 ± 0.21	0.03
Total cholesterol (mmol/l)	−0.08 ± 0.03	0.007
Log insulin (*μ*IU/mL)	−0.18 ± 0.09	0.04
Adjusted *R*^2^	0.11	
*p* value (final model)	<0.001	

Note: data presented as mean ± SE; *p* values<0.05 considered significant.

**Table 4:** Clinical characteristics of males and females according to studied groups.

Parameters	Males				Females			
	HL	OND	OD	*p* value	HL	OND	OD	*p* value
N (M/F)	41	46	40		44	54	55	
Age (years)	40.8 ± 14.3	41.8 ± 12.4^B^	49.1 ± 12.4^A^	0.009	37.0 ± 13.2	37.7 ± 4.5^B^	49.9 ± 8.9^A^	<0.001
BMI (kg/m^2^)	22.0 ± 2.2	35.5 ± 4.5^A^	34.2 ± 3.3^A^	<0.001	22.2 ± 2.1	36.2 ± 4.5^A^	34.8 ± 3.9^A^	<0.001
WHR	0.95 ± 0.12	0.94 ± 0.10^B^	1.02 ± 0.11	0.03	0.83 ± 0.1	0.85 ± 0.10^B^	0.90 ± 0.10^A^	0.004
SBP (mmHg)	116.4 ± 10.3	126.2 ± 11.5^A^	129.5 ± 11.8^A^	<0.001	111.3 ± 14.6	117.8 ± 9.6^AB^	128.8 ± 11.8^A^	<0.001
DBP (mmHg)	76.9 ± 6.4	81.1 ± 5.8^A^	82.4 ± 7.5^A^	0.002	71.7 ± 5.8	76.3 ± 5.6^AB^	81.4 ± 6.4^A^	<0.001
Glucose (nmol/L)	4.7 ± 0.6	4.8 ± 0.6^B^	10.9 ± 3.2^A^	<0.001	4.7 ± 0.6	4.9 ± 0.4^B^	11.8 ± 3.8^A^	<0.001
TC (nmol/L)	4.8 ± 1.0	5.1 ± 1.3	5.5 ± 1.8	0.08	4.6 ± 0.9	4.8 ± 0.9^B^	5.5 ± 1.4^A^	<0.001
HDL (nmol/L)	0.99 ± 0.3	0.89 ± 0.2	0.86 ± 0.3	0.09	1.2 ± 0.3	1.1 ± 0.3	1.0 ± 0.3^A^	<0.001
TG (nmol/L)	1.5 (1.1-2.0)	2.0 (1.2-2.6)^B^	2.5 (1.8-3.9)^A^	<0.001	1.0 (0.8-1.2)	1.4 (1-1.8)^AB^	2.0 (1.5-3.1)^A^	<0.001
Insulin (*μ*IU/mL)	4.1 (2.3-6.3)	9.9 (7-17.2)^AB^	17.5 (8-49)^A^	<0.001	3.0 (1.8-7.4)	7.3 (4-12)^AB^	12.0 (6-4)^A^	<0.001
HOMA-IR	0.82 (0.4-1.4)	2.3 (1.2-3.8)^AB^	8.8 (4-22)^A^	<0.001	0.7 (0.4-1.6)	1.5 (0.8-2.4)^B^	6.4 (2.6-13)^A^	<0.001
PLIN3 (ng/mL)	10.6 (2.2-43.6)	5.2 (0.9-35.2)	3.4 (0.9-17.6)	0.14	25.9 (9.5-36.6)	20 (7-27)^B^	9.7 (2.2-25)^A^	0.007

Note: data presented as mean ± SD for normal and median (interquartile range for nonnormal variables). Superscript “ indicates significant difference from HL, and superscript “B” indicates significance compared to OND. Significant at *p* < 0.05. BMI: body mass index; BP: blood pressure; DBP: diastolic blood pressure; HDL: high-density lipoprotein; HOMA-IR: homeostasis insulin resistance; TC: total cholesterol; TG: triglycerides; WHR: waist-hip ratio.

## Data Availability

The datasets used and/or analyzed during the current study are available from the corresponding author on reasonable request.
